# Clinical Outcomes after Mix-and-Match Implantation of Extended Depth of Focus and Diffractive Multifocal Intraocular Lenses

**DOI:** 10.1155/2021/8881794

**Published:** 2021-08-03

**Authors:** Jae Hyuck Lee, Ho Seok Chung, Su Young Moon, So Young Park, Hun Lee, Jae Yong Kim, Hungwon Tchah

**Affiliations:** ^1^Department of Ophthalmology, HanGil Eye Hospital, Incheon, Republic of Korea; ^2^Department of Ophthalmology, Dankook University Hospital, Dankook University College of Medicine, Cheonan, Republic of Korea; ^3^Department of Ophthalmology, Asan Medical Center, University of Ulsan College of Medicine, Seoul, Republic of Korea

## Abstract

**Purpose:**

To evaluate the clinical outcomes after bilateral mix-and-match cataract surgery using extended depth of focus (EDOF) and diffractive multifocal (DMF) intraocular lenses (IOLs). *Setting*. Asan Medical Center, Seoul, South Korea.

**Design:**

Prospective clinical study.

**Methods:**

Thirty-seven patients underwent TECNIS Symfony EDOF IOL (ZXR00) implantation in the dominant eye, and TECNIS +3.25 DMF IOL (ZLB00) implantation in the nondominant eye. Patients were followed up for 3 months; uncorrected and corrected distance visual acuity (UDVA and CDVA), uncorrected intermediate and near visual acuity (UIVA and UNVA), contrast sensitivity, defocus curves, stereopsis, and patient satisfaction were assessed.

**Results:**

At 3 months, the mean logarithm of the minimum angle of resolution (logMAR) of UDVA was 0.07 ± 0.09 in EDOF IOL eyes, 0.12 ± 0.11 in DMF IOL eyes, and 0.02 ± 0.05 in both eyes. UIVA was 0.11 ± 0.11 in EDOF IOL eyes, 0.16 ± 0.12 in DMF IOL eyes, and 0.04 ± 0.07 in both eyes. UNVA was 0.25 ± 0.15 in EDOF IOL eyes, 0.22 ± 0.16 in DMF IOL eyes, and 0.13 ± 0.13 in both eyes. Thirty patients (81.1%) were more than satisfied with near vision, and 8 patients (21.6%) complained of severe glare and halo. Spectacle independence for near vision was achieved in 34 patients (91.9%), and 31 patients (83.8%) had better than a 50-second arc of stereopsis.

**Conclusion:**

Mix-and-match cataract surgery with EDOF and DMF IOL implantation provided good visual outcomes for all distances. Additionally, excellent patient satisfaction was achieved with a high level of spectacle independence and acceptable photic phenomena.

## 1. Introduction

Until 20 years ago, cataract surgery using monofocal intraocular lenses (IOLs) could restore only uncorrected distance visual acuity (UDVA). However, due to accommodation loss, most patients must use reading glasses for near tasks [1]. Javitt et al. [2] found that only 9.8% of subjects studied who had undergone binocular monofocal IOLs surgery did not require reading glasses for viewing near targets and 80.4% needed reading spectacles for more than half of the time. Recently, binocular multifocal IOL surgery was proposed for cataract patients with presbyopia.

Diverse types of multifocal IOLs have been made for cataract patients requiring presbyopia treatment. Such IOLs achieved fine clinical results especially with proper patient selection [3, 4]. Currently, the most widely preferred multifocal IOLs in the market are diffractive and refractive lenses.

The multifocal TECNIS ZLB00 IOL (Johnson and Johnson Vision, Santa Ana, CA, USA), which has an anterior aspheric and posterior diffractive surface, creates two focal points and has a near addition of 3.25 diopters (D). The additional power of this IOL is relative to the IOL plane and is approximately 2.37 D at the spectacle plane. It distributes light evenly between the distance vision (42%) and the near vision (42%) regardless of the pupil size.

The new concept of extended depth of focus (EDOF) IOLs has recently been introduced for minimizing the photic phenomena frequently observed in refractive and diffractive multifocal IOLs of patients with a functional vision range. The standard aim of the TECNIS Symfony EDOF IOL (Johnson and Johnson Vision, Santa Ana, CA, USA) is to create a single elongated focal point for enlarging the depth of focus and thus offers a wide range of vision.

After the development of various multifocal IOLs, several multifocal IOLs have been manufactured for improving near visual acuity. Binocular implantation of different multifocal IOLs with various optic designs and near additions, the so-called mix-and-match method, was developed to take advantage of neural adaptation and thus accomplish improved visual outcomes [5, 6].

The purpose of the present study was to estimate postoperative visual acuities of various distances, refraction, photic phenomena, and patient satisfaction of those who underwent binocular cataract surgery with implantation of the TECNIS Symfony EDOF IOL (ZXR00) in the dominant eye and the +3.25 D TECNIS multifocal IOL (ZLB00) in the nondominant eye.

## 2. Materials and Methods

### 2.1. Study Design

This study was a single-center, prospective case series involving Korean patients who underwent cataract surgery at the Department of Ophthalmology, Asan Medical Center, Seoul, South Korea. All patients provided written informed consent prior to enrollment. The study followed the principles of the Declaration of Helsinki, was approved by the Asan Medical Center Review Board (2017-0713), and registered as a clinical trial (KCT0004111). This study basically shared similar platform with the study regarding mix-and-match implantation of diffractive multifocal IOLs with different diopter add powers performed by our institute [7].

Patients with cataracts aged 21 years or older, who had a potential distance visual acuity of 20/25 or better in each eye and a corneal astigmatism ≤1.50 D were included. The patients desired postoperative spectacle independence and were scheduled for binocular multifocal IOL implantations. Exclusion criteria included age older than 80 years, axial length (AL) of more than 26.0 mm, intraoperative complications, and any ocular diseases other than cataract.

Treatment was performed based on the mix-and-match approach as follows: the subjects received a TECNIS Symfony EDOF IOL (ZXR00) in the dominant eye and a TECNIS multifocal +3.25 D IOL (ZLB00) in the nondominant eye. The dominant eye was determined by the pinhole test and was implanted with the TECNIS Symfony EDOF IOL (ZXR00) [8]. One week after surgery of the dominant eye, the TECNIS ZLB00 was inserted in the nondominant eye. IOL power was calculated by formulating the corneal curvature and AL of the eye using an IOL Master 500 (Carl Zeiss Meditec AG, Jena, Germany). The patients' demographic and clinical data were recorded preoperatively.

The target refraction for the dominant eye was emmetropia. The target for the nondominant eye was dependent upon the patient's visual acuity and personal demands for precise near or distance vision, while also considering the postoperative refractive error (micro-monovision of ±0.3 D). After considering keratometry, AL, and the anterior chamber depth of each eye, the IOL power was chosen from the Sanders–Retzlaff–Kraff (SRK) II, Sanders–Retzlaff–Kraff/Theoretical (SRK/T), Haigis, or Hoffer Q formula.

### 2.2. Study Lenses

The TECNIS Symfony EDOF IOL (ZXR00) and the TECNIS multifocal +3.25 D IOL (ZLB00) IOL were used for this study. ZXR00 is a one-piece, biconvex, ultraviolet-blocking hydrophobic acrylic and pupil-independent diffractive IOL, which has anterior aspheric surface and a posterior achromatic surface with an echelette design for correction of chromatic aberrations, enhancement of contrast sensitivity, and the introduction of a novel pattern of light diffraction that elongates a single focal zone resulting in an extended depth of focus. ZLB00 is a bifocal, foldable hydrophobic acrylic one-piece IOL, which has 6.0 mm full-aperture diffractive optic with an overall diameter of 13.0 mm; it is an ultraviolet-blocking hydrophobic acrylic IOL with a full posterior diffractive multifocal optic surface and an anterior aspheric surface, has a near power of +3.25 D in the IOL plane, and it splits the light into two focal points for distance and near vision.

### 2.3. Surgical Technique

All surgeries were performed with the superior approach by a single experienced surgeon (H. Tchah). Topical anesthesia (proparacaine hydrochloride 0.5%) was administered; a continuous curvilinear capsulorrhexis (CCC) marker with a 6.0 mm diameter was used as a reference to the corneal plane, which was approximately 5.0 mm on the anterior capsule plane. The location of a 2.2 mm clear corneal incision was selected on the steepest meridian to minimize corneal astigmatism. A CCC and hydrodissection were then performed. Phacoemulsification and polishing were done, and an IOL was implanted into the capsular bag. All incisions were hydrated, and the surgery was finished without sutures. All patients were administered moxifloxacin and fluorometholone 0.1% eyedrops 4 times daily for 4 weeks postoperatively.

### 2.4. Outcome Measures

All subjects underwent comprehensive ophthalmological examinations preoperatively, including logarithm of the minimum angle of resolution (logMAR) visual acuity measurements of monocular and binocular UDVA, uncorrected intermediate visual acuity (UIVA), uncorrected near visual acuity (UNVA), and corrected distance visual acuity (CDVA). Preoperative assessments also included autorefraction and keratometry (Canon R-50; Canon USA Inc., Huntington, NY, USA), slit-lamp examinations (Haag-Streit, Köniz, Switzerland), biometry (IOL Master 500; Carl Zeiss Meditec, Jena, Germany), and corneal topography (Orbscan, Bausch and Lomb, Rochester, NY, USA).

The ophthalmic examinations conducted at 1 and 3 months after surgery included logMAR measurements of monocular and binocular UDVA, UIVA, UNVA, and CDVA. Autorefraction and keratometry were also performed. Intermediate visual acuity was measured at 60 cm. Near visual acuity was measured at 33, 40, and 50 cm, and the average visual acuity at these 3 distances was calculated. In most of the previous reports, near visual acuity was measured merely at 40 cm [9, 10]. However, we considered the various needs that exist for near distance in different situations such as reading newspapers and using smartphones; thus, we defined near visual acuity more widely in this study than is described above. In addition, monocular and binocular defocus curves were obtained at 3 months postoperatively with the measurement of monocular or binocular visual acuity at 4 meters ranging from distance correction, after which defocusing with added lenses in graded steps of 0.5 D from −4.00 D to 0.50 D.

Distance contrast sensitivity was measured in each uncorrected eye under photopic (85 cd/m^2^) and mesopic (3 cd/m^2^) conditions at 3 months postoperatively with the Functional Acuity Contrast Test function of the Ophtec 6500 view-in test system (Stereo Optical Co, Inc., Chicago, IL, USA) using 5 different points of stimulus spatial frequencies from 1.5 to 18 cycles per degree (1.5, 3, 6, 12, and 18). Stereopsis was evaluated using the Fly-S Stereopsis Test (Optimed, Sydney, Australia).

The patients completed a questionnaire regarding their overall satisfaction, occurrence of visual symptoms, and spectacle dependence for near and distant sight. Overall satisfaction was evaluated with a 5-point Likert scale: 1 = very dissatisfied, 2 = dissatisfied, 3 = neither satisfied nor dissatisfied, 4 = satisfied, and 5 = very satisfied. Visual symptoms (glare, halo, and visual disturbances at night or in the dark) were scored on a 5-point scale from 1 (absent symptoms) to 5 (very severe symptoms). Subjects also answered if they would recommend bilateral mix-and-match implantation of multifocal IOLs to their friends or relatives, with allowed responses being “yes” or “no.”

### 2.5. Statistical Analyses

Results are expressed as means ± standard deviations with ranges. The differences between preoperative and postoperative data were assessed using the Wilcoxon signed-rank test. The defocus curves for both the dominant eye and the nondominant eye were analyzed by the Kruskal–Wallis test with the Bonferroni correction. All statistical analyses were done with SPSS^®^ version 21 software (IBM, SPSS Inc., Chicago, IL, USA). *P* values less than 0.05 were considered statistically significant.

## 3. Results

In total, 74 eyes from 37 patients were included in this study, and all participants completed the 3-month postoperative visit. Preoperative subject and ocular characteristics are summarized in Table 1.

Intraoperatively, a radial tear occurred in one eye (1.4%) implanted with the TECNIS ZLB00 IOL. A posterior capsular rent developed in 2 eyes (2.7%) implanted with the TECNIS ZXR00 IOL, and an anterior vitrectomy was performed in these eyes because of vitreous loss. Nonetheless, the posterior capsular rent was not enlarged, and most parts of the posterior capsule were saved. Thus, IOLs could be implanted safely in the bag in those cases. No other complications occurred during the study.  Table 2 summarizes preoperative and postoperative monocular visual acuity. At 3 months postoperatively, monocular logMAR of UDVA, UIVA, and UNVA of the dominant eye were 0.07 ± 0.09 (range: 0–0.40), 0.11 ± 0.11 (range: 0–0.30), and 0.25 ± 0.15 (range: 0–0.40), respectively. Monocular logMAR of UDVA, UIVA, and UNVA of the nondominant eye were 0.12 ± 0.11 (range: 0–0.30), 0.16 ± 0.12 (range: 0–0.40), and 0.22 ± 0.16 (range: 0–0.40), respectively. Postoperative monocular logMAR of UDVA, UIVA, UNVA, and CDVAs were all significantly better than the preoperative values. Significant differences were also found between preoperative and postoperative binocular visual acuity logMAR of UDVA, UIVA, and l UNVA (Table 3). At 3 months postoperatively, binocular logMAR of UDVA, UIVA, and UNVA were 0.02 ± 0.05 (range: 0–0.20), 0.04 ± 0.07 (range: 0–0.30), and 0.13 ± 0.13 (range: 0–0.40), respectively.

**Figure 1:**
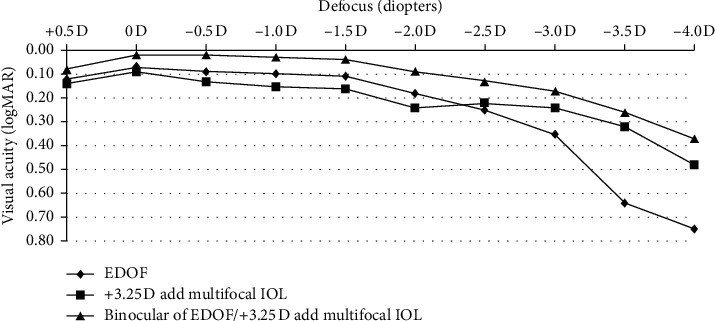
Binocular and monocular defocus curves of patients after bilateral mix-and-match implantation of extended depth of focus and diffractive multifocal intraocular lenses.

Binocular and monocular defocus curves are depicted in Figure 1. Eyes implanted with the diffractive multifocal IOL exhibited the expected bimodal peaks at 0 and −2.5 D. Eyes implanted with ZXR00 exhibited a visual acuity of 0.20 logMAR or better between 0 D and −2 D, although there was a sharp decrease in visual acuity over −2 D. The binocular defocus curve was better than each monocular defocus curve at all distances. Additionally, the binocular defocus curve exhibited nice visual acuity widely at all distances, as visual acuities were better than 0.20 logMAR between 0 D and −3 D.

**Figure 2:**
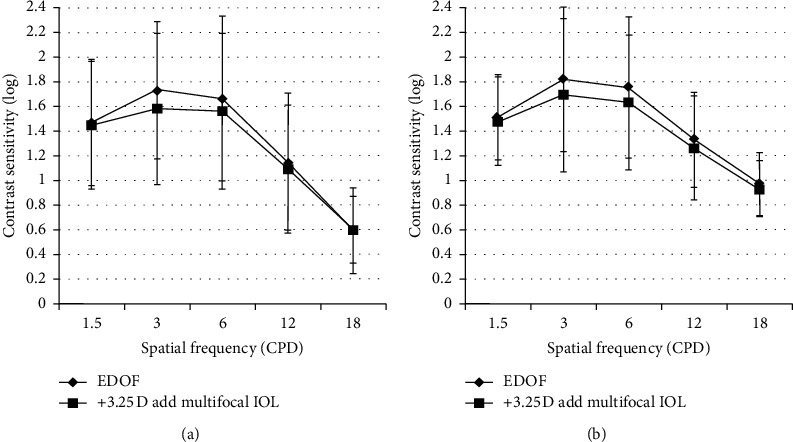
Contrast sensitivity test under photopic and mesopic conditions with mix-and-match implantation of extended depot of focus and diffractive multifocal intraocular lenses. CPD: cycles per degree. (a) Photopic. (b) Mesopic.

As shown in Figure 2, contrast sensitivities were evaluated in both photopic and mesopic situations in eyes implanted with EDOF and DMF IOLs and were not significantly different at any spatial frequency.

All subjects completed satisfaction questionnaires at 3 months postoperatively. Thirty subjects (81.1%) reported that they were satisfied or very satisfied with their near visual acuity, with an average satisfaction score of 4.0 ± 1.0 (range: 2–5). Only 3 subjects (8.1%) reported needing glasses occasionally for near vision after surgery. The rate of visual disturbances was low; 8 (21.6%) subjects reported glare and halo symptom scores worse than 3 (average score: 2.6 ± 1.0) (range: 1–5), and 31 (83.8%) patients exhibited better than a 50-second arc of stereopsis. Thirty-four subjects out of 37 (91.9%) answered that they would recommend mix-and-match implantation to their relatives (Table 4). No subjects experienced significant posterior capsular opacity or dislocated IOL 3 months postoperatively.

## 4. Discussion

Currently, various IOLs and surgical approaches are suggested for cataract surgery and presbyopia correction including monovision accommodating IOLs, multifocal IOLs, and the mix-and-match method. However, all of these IOLs and approaches have shortcomings and weaknesses [11–13]. One study using multifocal IOLs reported that subjects were content with a unilaterally implanted IOL in the dominant eye; however, the bilateral approach showed better visual acuities and enhancement of stereopsis [14].

Gunenc and Celik [6] speculated that using one kind of multifocal IOL might not offer a full span of vision and therefore suggested contralateral insertion of two different kinds of multifocal IOLs with cataract surgery using refractive and diffractive multifocal IOLs.

The TECNIS Symfony EDOF IOL (ZXR00) has shown to offer good far, intermediate and near visual outcomes, and better outcomes especially for far and intermediate visual acuities, but with a few limitations in near visual acuity [15]. However, compared with other multifocal IOLs, ZXR00 results in fewer postoperative photic phenomena [16]. In this study, with the strength in photic phenomena, the limitation of decreased near visual acuity in EDOF IOLs was overcome by mix-and-match implantation of a DMF IOL in the nondominant eye. TECNIS +3.25 DMF IOLs (ZLB00) were selected, as they demonstrated the best near visual acuities among various types of DMF IOLs in a recent study [17].

In the present study, the multifocal IOL mix-and-match approach bilaterally achieved excellent binocular UDVA, UIVA, and UNVA outcomes over the follow-up period of 3 months. Binocular uncorrected visual acuity showed a wide range of good postoperative vision across all distances. This is considered an adequate outcome, as this approach enabled subjects to read the books or use smartphones without glasses. Binocular uncorrected visual outcome was relatively better compared to monocular outcomes at all ranges in both groups, and it can be supposed that this is a result of bilateral summation, which was presented in the previous study [18].

Even though postoperative satisfaction declined partially with the passing of time, approximately 80% of subjects answered that they were moderately satisfied or more 3 months postoperatively. The main cause of dissatisfaction was photic phenomena, as reported through the questionnaire. And, the rate of severe glare and halo was similar to that of “not satisfied” as approximately 20%. Photic phenomena are usually the main reasons for multifocal IOL explantations [19]. In the present study, the proportion of patients that reported severe photic phenomena was quite low (approximately 20%). However, no case required IOL explantation, and the rate of severe photic phenomena was comparable with those reported in previous studies [15, 20, 21]. Furthermore, over 90% of the subjects answered that they would recommend this type of surgery to relatives or friends. Moreover, over 80% of patients were able to obtain sufficient near stereopsis better than a 50-second arc after surgery without spectacles; this is an additional advantage of mix-and-match cataract surgery.

There are some limitations to this study that should be acknowledged. The small number of cases and relatively short-term follow-up duration warrant for further larger and longitudinal studies. In addition, the incidence of photic phenomena was only subjectively evaluated due to the absence of appropriate device to evaluate visual symptoms more properly. Finally, in the first three patients, the dominance examination was not performed before surgery; therefore, the possibility of EDOF IOL implantation in the nondominant eye in these patients cannot be ruled out. However, eye dominance is changeable; therefore, the effect in this study would not have been significant.

## 5. Conclusions

Mix-and-match cataract surgery with EDOF and DMF IOL implantation provided good uncorrected near, intermediate, and distance visual acuity 3 months postoperatively with acceptable photic phenomena and favorable patient satisfaction. This surgery is expected to improve patient satisfaction as it lowers dependence on glasses without causing serious photic phenomena.

## Figures and Tables

**Table 1:** Demographic and clinical characteristics of the patients.

Number of eyes/patients	74/37
Sex (male/female)	19/18
Age (years)	64.55 ± 6.73 (range: 50–79)
Mean corneal astigmatism (D)	0.63 ± 0.31 (dominant eye) (range: 0–1.38), 0.75 ± 0.46 (nondominant eye) (range: 0–1.50)
Mean spherical equivalent (D)	0.63 ± 2.19 (dominant eye) (range: −5.75–4.00), 0.44 ± 2.37 (nondominant eye) (range: −6.00–3.50)
Mean axial length (mm)	23.58 ± 0.96 (dominant eye) (range: 21.43–25.82), 23.65 ± 0.99 (nondominant eye) (range: 21.46–25.99)

Results reported as means ± standard deviations. D = diopters.

**Table 2:** Refractive outcomes and monocular visual acuity in patients who underwent mix-and-match implantation of an extended depth of focus and a +3.25 D near add diffractive multifocal intraocular lens.

		LogMAR UDVA	LogMAR UIVA	LogMAR UNVA	LogMAR CDVA
Preoperative	Dominant eye	0.40 ± 0.25 (range: 0–0.80)	0.53 ± 0.26 (range: 0.20–1.00)	0.57 ± 0.23 (range: 0.20–1.00)	0.19 ± 0.20 (range: 0–0.50)
	Nondominant eye	0.45 ± 0.31 (range: 0.10–0.90)	0.55 ± 0.23 (range: 0.20–1.00)	0.67 ± 0.23 (range: 0.30–1.00)	0.17 ± 0.14 (range: 0–0.50)

1-month postoperative	Dominant eye	0.11 ± 0.09 (range: 0–0.30)	0.14 ± 0.11 (range: 0–0.30)	0.26 ± 0.18 (range: 0–0.50)	0.02 ± 0.06 (range: 0–0.10)
	*P* value^*∗*^	<.001	<.001	.011	<.001
	Nondominant eye	0.12 ± 0.11 (range: 0–0.30)	0.18 ± 0.13 (range: 0–0.50)	0.21 ± 0.13 (range: 0–0.50)	0.04 ± 0.05 (range: 0–0.20)
	*P* value^*∗*^	<.001	<.001	<.001	<.001

3-month postoperative	Dominant eye	0.07 ± 0.09 (range: 0–0.40)	0.11 ± 0.11 (range: 0–0.30)	0.25 ± 0.15 (range: 0–0.40)	0.02 ± 0.05 (range: 0–0.10)
	*P* value^*∗*^	<.001	<.001	.009	<.001
	Nondominant eye	0.12 ± 0.11 (range: 0–0.30)	0.16 ± 0.12 (range: 0–0.40)	0.22 ± 0.16 (range: 0–0.40)	0.04 ± 0.07 (range: 0–0.20)
	*P* value^*∗*^	<.001	<.001	<.001	<.001

Results reported as means ± standard deviations. UDVA: uncorrected distance visual acuity; UIVA: uncorrected intermediate visual acuity; UNVA: uncorrected near visual acuity; CDVA: corrected distance visual acuity. ^*∗*^Compared with preoperative values.

**Table 3:** Binocular visual acuity in patients with mix-and-match implantation of an extended depth of focus and a +3.25 D near add diffractive multifocal intraocular lens.

	Preoperative	1-month postoperative	*P* value^*∗*^	3-month postoperative	*P* value^*∗*^
LogMAR UDVA	0.25 ± 0.19 (range: 0–0.80)	0.02 ± 0.04 (range: 0–0.10)	<.001	0.02 ± 0.05 (range: 0–0.20)	<.001
LogMAR UIVA	0.37 ± 0.18 (range: 0.10–0.80)	0.05 ± 0.06 (range: 0–0.20)	<.001	0.04 ± 0.07 (range: 0–0.30)	<.001
LogMAR UNVA	0.49 ± 0.22 (range: 0.10–0.80)	0.13 ± 0.11 (range: 0–0.40)	<.001	0.13 ± 0.13 (range: 0–0.40)	<.001
LogMAR CDVA	0.09 ± 0.08 (range: 0–0.30)	0.00 ± 0.01 (range: 0–0.10)	<.001	0.00 ± 0.02 (range: 0–0.10)	<.001

Results reported as means ± standard deviations. UDVA; uncorrected distance visual acuity; UIVA: uncorrected intermediate visual acuity; UNVA: uncorrected near visual acuity; CDVA: corrected distance visual acuity. ^*∗*^Compared with preoperative values.

**Table 4:** Questionnaire results regarding overall satisfaction, visual symptoms, and spectacle dependence.

Questionnaire	Response (average score/rate)
Overall satisfaction	4.0 ± 1.0 (range: 2–5), very satisfied or satisfied: 81.1% (30 patients)
Needing for near glasses after surgery	3.9 ± 1.2 (range: 3–5), occasionally need near glasses: 8.1% (3 patients)
Glare and halo symptoms	2.6 ± 1.0 (range: 1–5), over score 3: 21.6% (8 patients)
Stereopsis	37.6 ± 15.5 (range: 20–63), better than 50 s: 83.8% (31 patients)
Recommendation for mix-and-match implantation	Yes: 91.9% (34 patients)

Results reported as means ± standard deviations. IOL: intraocular lenses. Satisfaction scale; 5 = very satisfied; 1 = very dissatisfied; need for near glasses; 5 = not at all; 1 = always needed. Scale of discomfort due to visual symptom: 5 = very severe symptoms; 1 = absence of symptoms. Stereopsis: seconds of arc.

## Data Availability

The data used to support the findings of this study are available from the corresponding author upon request.
